# The price of nicotine dependence: A comparison of the cost of nicotine across products in Switzerland, Germany, USA, Sweden, France and the UK, in 2019

**DOI:** 10.18332/tpc/156052

**Published:** 2022-11-25

**Authors:** Julian Jakob, Sandra Joss, Armando N. Meier, Kali Tal, Anna Schoeni, Joachim Marti, Pascal Diethelm, Reto Auer

**Affiliations:** 1 Institute of Primary Health Care (BIHAM), University of Bern, Bern, Switzerland; 2 Department of Pediatrics, University Hospital Bern, Inselspital, Bern, Switzerland; 3 Graduate School for Health Sciences, University of Bern, Bern, Switzerland; 4 Centre for Primary Care and Public Health (Unisanté), University of Lausanne, Lausanne, Switzerland; 5 OxySuisse, Geneva, Switzerland

**Keywords:** health economics, smoking cessation, tobacco, nicotine replacement, health equity

## Abstract

**INTRODUCTION:**

Tobacco cigarette taxes aim at reducing smoking, but smokers are still dependent on nicotine and need safe and cheap alternatives. As the costs play a role in the product chosen, we compared standardized nicotine costs across products and countries.

**METHODS:**

We gathered prices of tobacco cigarettes, heated tobacco products (HTP), pharmaceutical nicotine replacement therapy (pNRT) gums, snus, and open and closed electronic nicotine delivery systems (ENDS) in 6 countries (Switzerland, Germany, USA, Sweden, France, UK) in 2019. We compared the cost of a pack of cigarettes in Switzerland to the cost of equivalent doses of nicotine delivered by other products and across countries, normalizing to purchasing power GDP per capita to compute relative adjusted costs (RACs).

**RESULTS:**

Adjusted tobacco cigarette cost was lowest in Switzerland, Germany, and Sweden; RAC for pNRT was 1.1 in Switzerland and 1.0 in Germany. In France and the UK, RACs for cigarettes were 1.5 and 2.1, while for pNRT they were cheaper (RAC: 0.04). In Switzerland, snus/nicotine pouches were the cheapest form of nicotine delivery (RAC: 0.2), open ENDS were a low-cost option for nicotine delivery in all countries (RAC: 0.2–0.3), and HTP cost more than regular tobacco products in most countries.

**CONCLUSIONS:**

We found broad differences in costs of nicotine according to countries and products. This should be considered in future studies on smoking prevalence and in public health efforts.

## INTRODUCTION

In Switzerland and Germany, about 30% of the adult population smokes^1,2^. Smokers who want to quit but are addicted to nicotine can use smoking cessation aids, such as pharmaceutical nicotine replacement therapy (pNRT, e.g. nicotine gums), that are safe and effective^3^, but smokers may be discouraged from taking NRT if the therapy costs more than cigarettes.

Pharmaceutical NRT prices vary across countries and only a few health systems reimburse their cost, as in France and in the UK. If pNRT cost too much, as in Switzerland or Germany, smokers may use other, potentially cheaper nicotine products like heated tobacco products (HTP), snus (traditional use in Sweden) or nicotine pouches, or electronic nicotine delivery systems (e-cigarettes or ENDS). HTP, hybrid devices that heat tobacco electronically, are heavily marketed by the tobacco industry as safer alternatives to traditional cigarettes, but still release smoke and have been shown to be associated with health risks compared to quitting^4^. Snus contains tobacco, whereas nicotine pouches are tobacco-free. Both are collocated orally between the gingiva and the cheek wall, where they release nicotine absorbed directly by mucosal tissue. ENDS are increasingly popular devices that heat a mixture of glycerin, propylene glycol, aromas, and optionally, nicotine, and emit an aerosol that contains much lower amounts of toxic compounds than cigarette smoke^5^. Some ENDS are ‘open system’ (the users add their own liquids) and some are closed (disposable pods contain premixed liquids). ENDS may help smokers quit but most clinical guidelines do not recommend them yet as these products have not been on the market for long, so they are not covered or reimbursed in most health systems^6^.

Smokers seeking to quit must often choose between recommended but more costly pNRT and cheaper, more accessible, but often less safe alternative cessation aids. The cost of alternatives to tobacco smoking varies across countries, but we do not know by how much. We found no previous study which compared price of tobacco-based and non-tobacco-based nicotine products across countries. This manuscript documents price differentials by collecting price data across 6 countries, adjusting for nicotine bioavailability and purchasing power.

## METHODS

### Information source

We gathered information on the price of tobacco cigarettes, heated tobacco products (HTP, e.g. IQOS®), pharmaceutical NRT gums, and open and closed ENDS in 2019 via the data portal of Euromonitor International Ltd, a London-based market research company. To confirm reliability, we compared tobacco cigarette price from Euromonitor with prices reported by the WHO^7^. Our study included Switzerland, Germany (both with lose tobacco control policies and expensive pNRT), the USA (high proportion of ENDS users), Sweden (traditional snus users), France, and the UK (strong tobacco control policies). We assessed the price of snus/nicotine pouches at the sites of online retailers in countries where these are legal.

### Nicotine content, bioavailability, and affordability

We collected data on the prices of the cheapest and most expensive brands of tobacco cigarettes, HTP, snus/nicotine pouches, pNRT 2 mg gums, and open and closed ENDS (devices and e-liquids) in each country. Then, we used mean prices to compare the relative doses of nicotine these products delivered; our reference was the mean price of a pack of cigarettes in Switzerland (=1), containing 20 cigarettes. We conservatively assumed that one average tobacco cigarette delivers 1 mg of nicotine to the smoker, since tobacco leaves, brands, additives, and inhalation modes vary^8^. We calculated that one pack of cigarettes delivers 20 mg of nicotine. Based on the literature, we assumed that tobacco cigarettes and open and closed ENDS deliver the same percentage of bioavailable nicotine (100%)^9,10^, but that HTP deliver 80% of the bioavailable nicotine that cigarettes deliver^10,11^. We assumed pNRT gums deliver 65%^12^, and snus/nicotine pouches 30%. We calculated what it would cost users to absorb 20 mg nicotine (nicotine delivered × 1/bioavailability). We estimated that ENDS user inhales around 3.5 mL of liquid per day, and added 20% of the liquid’s price to account for initial device costs and coils (Supplementary file). We added 10% of the price of tobacco sticks (HTP)/liquids pods (closed ENDS) to account for initial device costs, as stick/pods are more expensive than e-liquids used in open ENDS. In countries where programs give smokers discounted or free pNRT to help them quit (USA, UK, France, Sweden), we set the adjusted cost to the user as an out-of-pocket expense of 10% of the original price. Finally, we adjusted prices across countries based on purchasing power adjusted GDP per capita (ppGDPpc), as done in other studies on tobacco affordability^13,14^. We calculated relative adjusted cost (RAC) as the price of 20 mg absorbed nicotine × ppGDPpc. We made all calculations in US$ in Microsoft Excel.

## RESULTS

**Figure 1:**
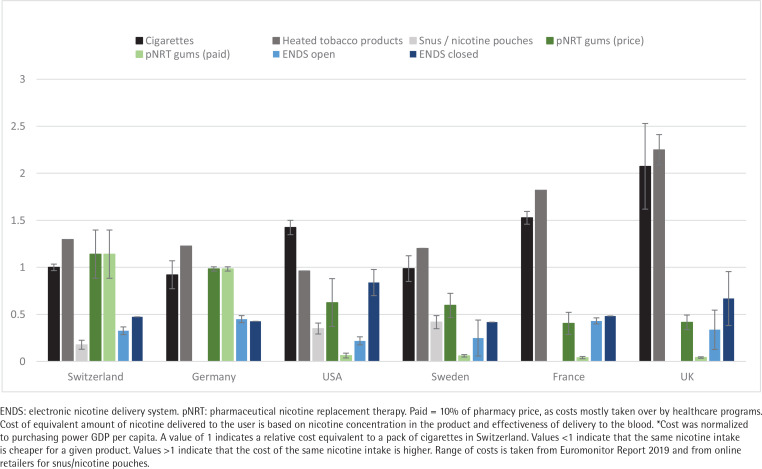
Relative adjusted costs of nicotine products across countries in 2019, compared to the price of one pack of tobacco cigarettes in Switzerland: purchasing power adjusted GDP per capita and bioavailability adjusted*

Unadjusted prices for a pack of cigarettes varied between $8.40 and $9 in Switzerland. Other unadjusted prices are presented in Supplementary file Table 1. Sweden’s prices were lowest ($5.60) and the UK’s highest ($15). Relative adjusted costs (RACs) for cigarettes were lowest in Switzerland, Germany and Sweden (1.0, 0.9, and 1.0, respectively) (Figure 1). RAC for pNRT was 1.1 in Switzerland, 1.0 in Germany (not covered by health insurance in both countries) and 0.6 in Sweden. In France, RAC for cigarettes was 1.5, and in the UK 2.1, but health insurances covered pNRT and patients paid very little out-of-pocket (RAC: 0.04). Even without accounting for health insurance coverage or tobacco cessation programs, pNRT costs much less in France and the UK than in Switzerland or Germany. In Switzerland, snus/nicotine pouches were the cheapest form of nicotine delivery (RAC: 0.2). Open ENDS were inexpensive in all countries (RAC: 0.2–0.4), while closed ENDS were more expensive (RAC: 0.4–0.8). HTP cost more than regular tobacco products in most countries (RAC: 1.0–2.2). Our results were similar when we used WHO reported prices for tobacco cigarettes.

## DISCUSSION

Switzerland and Germany had the lowest relative adjusted cigarette cost (RAC); they were close to the RAC of pNRTs. In the UK and France, RAC of cigarettes was up to 2 times higher than in Switzerland, while pNRTs were 10 times cheaper than cigarettes in these countries. Open ENDS, and snus/nicotine pouches (where legal), were cheap forms of nicotine delivery in all countries, while closed ENDS were cheaper than cigarettes, but more expensive than open systems. Heated tobacco products were more expensive than cigarettes in most countries.

Large differences in costs of nicotine products are likely to lead to differences in use of products. The UK and France follow a public health strategy aiming at guiding smokers to quit via pNRT, with high cigarette prices and a healthcare system that reimburses pNRT, while pNRTs are cheap even without insurance or smoking cessation program. In other countries, where pNRTs are unaffordable alternatives for smoking cessation, highly nicotine-dependent smokers may turn to cheaper products like snus/nicotine pouches or open ENDS. Thus, smokers wanting to quit must choose between officially recommended expensive pNRT and newer products that might be effective but are not part of an official public health smoking cessation strategy, with potential unknown health hazards. Several randomized controlled trials report on the effectiveness of ENDS for smoking cessation^6^. Evidence is weaker for snus/nicotine pouches^15^, but in Sweden, levying a lower excise tax on snus than on cigarettes, making the smokeless product cheaper and giving it a competitive advantage, snus is used by 25% of men, while less than 10% of Swedish men smoke^16^. For now, only the UK officially includes ENDS in their harm reduction strategy, and Sweden includes snus.

We know that lower income smokers are more price sensitive^17^. Especially for this population, keeping the costs of nicotine replacement products reasonable is a necessary component of tobacco harm reduction strategies. Prices could be kept low if governments exerted strong market control and set pNRT prices or if public healthcare insurances covered the costs of pNRTs, which are recommended smoking cessation medications. This has happened in France. Besides lowering out-of-pocket expenses for smokers directly, retail selling prices of pNRT decreased significantly as well, once reimbursed by health insurance.

### Limitations

Our study has some limitations. Snus/nicotine pouches were illegal in some jurisdictions, so prices were not reported in Euromonitor, so we might have missed effects in settings with high shares of products from the illicit markets. Also, as Euromonitor might not be independent from the tobacco industry, we cannot be fully confident in the accuracy of prices reported. To account for that, we compared prices with statistics provided by WHO, and found similar results. We did not account for variance of income and insurance coverage in countries but focused on mean prices and adjusted costs across nicotine products and countries. Our study should thus be considered as a first pilot attempt to compare cost of nicotine products across a limited number of countries. We hope future research will extend the comparison among more countries with more refined methods.

## CONCLUSIONS

This study provides descriptive evidence of broad variations in costs of nicotine products. Scientists should further study the effectiveness and toxicology of nicotine products to enable implementation of a reasonable cost-benefit analysis of these products. Future studies should track associations between cost of tobacco and nicotine products and smoking prevalence and rate.

## Data Availability

The data supporting this research can be found in the Supplementary file.
